# Renal Transplantation from Elderly Living Donors

**DOI:** 10.1155/2013/475964

**Published:** 2013-09-12

**Authors:** Jacob A. Akoh, Umasankar Mathuram Thiyagarajan

**Affiliations:** South West Transplant Centre, Plymouth Hospitals NHS Trust, Derriford Hospital, Plymouth PL6 8DH, UK

## Abstract

Acceptance of elderly living kidney donors remains controversial due to the higher incidence of comorbidity and greater risk of postoperative complications. This is a review of publications in the English language between 2000 and 2013 about renal transplantation from elderly living donors to determine trends and effects of donation, and the outcomes of such transplantation. The last decade witnessed a 50% increase in living kidney donor transplants, with a disproportionate increase in donors >60 years. There is no accelerated loss of kidney function following donation, and the incidence of established renal failure (ERF) and hypertension among donors is similar to that of the general population. The overall incidence of ERF in living donors is about 0.134 per 1000 years. Elderly donors require rigorous assessment and should have a predicted glomerular filtration rate of at least 37.5 mL/min/1.73 m^2^ at the age of 80. Though elderly donors had lower glomerular filtration rate before donation, proportionate decline after donation was similar in both young and elderly groups. The risks of delayed graft function, acute rejection, and graft failure in transplants from living donors >65 years are significantly higher than transplants from younger donors. A multicentred, long-term, and prospective database addressing the outcomes of kidneys from elderly living donors is recommended.

## 1. Introduction

Kidney transplantation is the optimum replacement therapy for patients with established renal failure (ERF), as it offers better quality of life and improved survival [1]. The demand for renal transplantation has increased due to the growing prevalence of ERF and extension of the criteria for accepting patients onto the waiting list. In response to the increasing need for organs, deceased donor programs (donation after circulatory death (DCD) and donation after brain death (DBD)) are being optimized, and living kidney donation expanded in several countries to include both related and unrelated donation. Further developments include ABO (blood group) incompatible transplantation, legalised altruistic nondirected living donation, and adoption of paired or more complex exchange of living donor programs. In the last decade in the UK, there has been significant growth in living donor kidney transplantation with 485 transplants in 2005, increasing to 1,055 in 2012 [2]. All these have not succeeded in meeting the demand for renal transplantation, and efforts to provide more donors have included the use of marginal living donors, particularly elderly living donors.

The use of grafts from elderly deceased donors (DD) is associated with less than ideal graft function and graft survival in recipients [3, 4]. This is attributed to reduced nephron mass, senescence, greater susceptibility to ischemic injury, and acute rejection episodes being more prevalent in the elderly. Whereas donor age is a strong determinant of death censored graft survival among recipients of deceased donor kidneys [5], the relationship between living donor age and graft survival is less clear [6, 7]. Kidneys from deceased older donors are more likely to be transplanted into older recipients, and this may potentially confound the impact of donor age on outcome [8]. Acceptance of elderly living donors remains controversial due to the higher incidence of comorbidity and greater risk of postoperative complications [9]. Such a risk to a donor whose benefit is at best psychological may be difficult to justify, as it may stretch the “first do no harm principle” [5–8].

As waiting times for transplantation increase, older candidates are more disadvantaged by their rapidly deteriorating health, often resulting in death or removal from the waiting list before transplantation [10]. UK transplant statistics for 2011/2012 show that 18% of patients on the transplant waiting list have either died or been removed from the waiting list by five years of listing [2]. Furthermore, only 65% of patients listed would have received a transplant by five years. This is a review of published evidence about renal transplantation from elderly living donors to determine trends in elderly living donation, the effect of donation on elderly donors, and outcomes of such transplantation.

## 2. Methodology

English language publications on living donor renal transplantation from 2000 to 2013 were obtained from electronic databases such as MEDLINE, The Cochrane Central Register of Controlled Trials, EMBASE (2000–2012), the database of abstracts of reviews of effects (DARE), OVID System, health technology assessment (HTA) Database, Google Scholar, and Elsevier's scientific search engine (SCIRUS); pertinent journals were searched to identify relevant studies including randomizes trial, meta-analysis, and case series.

### 2.1. Search Strategy

The search strategy included but not limited to the terms “kidney,” “renal,” “transplantation,” “older,” “living,” “donor,” “outcomes,” “graft survival,” and “patient survival.” These key words were used in combination with their corresponding subject headings. Duplicate articles were excluded on the basis of abstract review. All related referenced articles in the English language literature between 2000 and 2013 were reviewed, and studies involving combined renal and other solid organ transplantation were excluded. All potential and relevant citations were retrieved in full text for detailed evaluation. When the same group of donors was studied in multiple publications, all were reviewed, and the strongest evidence-based article with long-term followup was cited.

## 3. Effects of Aging on Kidney Function

The number of glomeruli per kidney and the mean glomerular volume negatively correlate with age beyond 60 years but positively correlate with kidney weight [11]. The number of sclerosed glomeruli per kidney also increased to 30–50% after the age of 60 [11, 12]. Humans may lose renal reserve as they age because of nephron loss, possibly secondary to glomerulosclerosis and/or renal microvascular disease [13]. In healthy nonnephrectomised individuals, induced renal hyperfiltration varies between 20, and 35% of basal nonstimulated glomerular filtration rate (GFR) [14]. Although the postnephrectomy GFR may not be affected by age, the post nephrectomy reserve capacity of the remaining kidney as assessed by amino acid-induced hyperfiltration was significantly impaired in older and heavier donors [14].

Kidneys from elderly donors have a lower functional nephron mass at the time of transplantation, compared with allografts from younger donors [15]. Following kidney donation however, there is no accelerated loss of kidney function [16]. The short-term kidney function in the donor recovers to 70% of predonation estimated GFR [17]. The incidence of ERF among donors is similar to that of the general population and ranges from 0.2 to 0.6% [18, 19].

The process of aging results in complex alterations in how the kidney copes with normal homeostasis as well as acute and chronic injury. Distinct processes that can explain why the kidney from an elderly donor regenerates less satisfactorily than the kidney from a younger donor include a diminished proliferative reserve, which might in part depend on altered progenitor cell function, an increased tendency for apoptosis, alterations in growth factor profiles, and important changes in immune responses [20, 21]. According to the 2006 United States Renal Data System report, the risk of DGF in transplants from living donors above the age of 65 years is double that of transplants from younger donors [22]. Another report, a retrospective cohort study of 49,589 recipients between 2000 and 2009 in the United States, showed that for every 10-year increase in living donor age, the odds of DGF increased by 15% [23]. These observations can be partly explained by the impaired ability for repair in kidneys from elderly donors [24].

## 4. Criteria for Selection of Living Donors

The goal of living donor assessment is to ensure the suitability of the donor and minimisation of risk of complications [25, 26]. The benefits to both donor and recipients must outweigh the risk associated with donation [27]. A key component of donor selection is a comprehensive medical assessment according to the criteria set by the Amsterdam forum [28]. This is in line with the procedure advocated by the British Transplantation Society (BTS) [29]. During assessment, the donor should be considered to be a patient just like the transplant recipient and should get the same level of care and protection against risks [30]. Elderly donors require even more rigorous assessment due to the higher prevalence of co-morbid conditions.

### 4.1. Glomerular Filtration Rate (GFR)

Living kidney donors with normal renal function prior to donation are at no greater risk of developing ERF after unilateral nephrectomy than individuals in the general population [18, 31]. A direct measurement of GFR using iodinated or radioactive isotopes is ideal for assessing renal function in a potential donor. However, most transplant centres determine GFR by measuring creatinine clearance using a 24-hour urine collection [32].

Several studies recommend that living donors should have a GFR of  ≥80 mL/min or, alternatively, a normal kidney function level within two standard deviation, (SD) for age and gender [26, 33, 34]. However, some centres utilise a GFR of 80 mL/min per 1.73 m^2^ to be the lower limit for donation [34–36]. There are relatively few published data on kidney function in normal populations stratified by age. The use of a single cutoff value does not take into account the decline in GFR with aging [37, 38]. Therefore, to identify potential donors at increased risk of developing ERF, cutoffs should vary based on age—on the premise that an individual would not develop clinically significant renal impairment as a result of unilateral nephrectomy. Most of the centres that utilise a cut-off GFR do so on what the likely GFR would be at the age of 80 [29]. Whether this means that, after 80, a donor might be accepted with an even lower GFR is debateable. But Nordén et al. [39] had shown that transplants from living donors with a GFR of 80 mL/min (not adjusted for body surface area) had graft survival worse than those from donors with higher GFR. Furthermore, graft survival was similar to figures reported for transplants from deceased donors. This highlights the fact that the evaluation of kidney function is important not only to protect the health of a donor, but also to ensure adequate function for the recipient.

Measurement of eGFR in living donors has not been validated to predict the risk of long-term kidney disease and should not be used in this context. The BTS guideline recommends that a prospective donor should not be considered for donation if the corrected GFR is predicted to fall below a satisfactory level of kidney function within the lifetime of the donor. For example, a predicted GFR of at least 37.5 mL/min/1.73 m^2^ at the age of 80 is recommended as a minimum standard [29]. There is a lack of evidence to guide acceptable levels of kidney function for donors over 60 years of age.

### 4.2. Hypertension

Brindel et al. [40] conducted a population-based study of noninstitutionalised individuals aged ≥65 years and reported that 62% of 9090 people were hypertensive with 81% of these on antihypertensive drugs. This finding is similar to another study using the database of the National Health and Nutrition Examination Survey [41], which showed the prevalence of hypertension (defined as ≥140/90 mmHg or taking antihypertensive medications) to be 7.3 ± 0.9%, 32.6 ± 2.0%, and 66.3 ± 1.8% in the 18 to 39, 40 to 59, and ≥60 age groups, respectively. The Framingham study showed that hypertension was more common in the elderly, and the overall risk for a cardiovascular events and deaths due to cardiovascular disease was two to three times higher in subjects with definite hypertension compared with normotensives for all age and sex groups considered [42].

Mild to moderate hypertension that is controlled with single or double antihypertensive agents is not a contraindication to kidney donation providing significant end-organ damage has been excluded [2]. The presence of hypertensive end organ damage, poorly controlled hypertension, or hypertension that requires more than two drugs to achieve adequate control are relative contraindications to living donation.

### 4.3. Other Medical Comorbidities

Evaluating the potential medical risks to individual donors presents a complex problem, particularly in the elderly, where age-associated conditions such as the decline in GFR, hypertension, impaired glucose tolerance, and weight gain assume increased significance after nephrectomy. In assessing people for kidney donation, postnephrectomy risks should be evaluated in terms of exposure over the duration of remaining lifetime. The Mayo kidney/pancreas transplant program stratifies medical criteria according to age, allowing more liberal criteria for older donors—based on their belief that many of the long-term results of kidney donation are likely to hinge upon future behaviour, including smoking, weight management, and medical follow-up care. Older donors are more likely to have established behavioural patterns, an element that makes them better candidates in many respects [43]. Such an approach requires careful follow up in order to determine the impact of donor nephrectomy in the current evolving environment.

Studies addressing the issue of accelerated kidney damage in patients with single kidneys who develop diabetes have produced conflicting results [44]. It is thought that hyperfiltration of the remaining kidney would cause donors who develop diabetic nephropathy to accelerate to ERF more rapidly than patients with two kidneys, but there is no evidence in donors who develop diabetes to prove this hypothesis. However, those who develop diabetes after kidney donation do have more proteinuria and hypertension [45]. Many guidelines, such as the one by the Amsterdam forum [28], while recommending that individuals with diabetes should be excluded from donating, they do not address donors in the prediabetes state. On the basis of cohort studies, ERF in living donors, a rare event, occurs in a median of 20 years after donation [31]; therefore, the younger the potential donor with impaired fasting glucose, the greater the cumulative risk for developing diabetes and its resultant complications. The observation by Vigneault and coworkers [44] that younger patients with prediabetes have more time to develop diabetes and its complications would imply that exclusion of older donors with impaired glucose tolerance could be relaxed, as the interval to developing diabetic nephropathy would make it irrelevant, as most elderly donors would have died from other causes.

## 5. Trends in Living Donation

Data from 2006 indicate that the greatest number of living donor kidney transplants were performed in the United States (6,435), United Kingdom (2,020), Brazil (1,768), Iran (1,615), Mexico (1,459), and Japan (939). During the last decade, 62% of the countries reported at least a 50% increase in the number of living kidney donor transplants. Also, the number of living donor kidney transplants performed in the US and Canada doubled and represented about 40% of all donor kidneys [46]. Horvat et al. [47] reported that about 27,000 related and unrelated living donor kidney transplants were performed worldwide in 2006, representing 39% of all kidney transplants. This growing trend is also reflected by the increase in the number of living donors >60 years during the last decade [48].

Since 2007, there have been more living donor transplants performed in the UK than deceased donor transplants. Whereas 34% of deceased donors in the UK were aged over 60 years, the proportion of living donors aged over 60 in 2011/2012 was 14% (NHS Blood and Transplant—personal communication). Furthermore, the characteristics of the living donors have changed, particularly with the acceptance of progressively older living donors [49].

## 6. Effect of Uninephrectomy on Donors

### 6.1. Complications in Elderly Donors

Friedman et al. [50] reviewed 6320 cases of living donors and reported a complication rate of 18.4% with no mortality. Independent predictors of donor complications were older age (odds ratio (OR), 1.01), male sex (OR, 1.19), Charlson comorbidity index of at least 1 (OR, 1.49), obesity (OR, 1.76), medium-size hospitals (OR, 1.88), and low-volume hospitals (OR, 1.37). Other factors affecting donor risk of chronic kidney disease (CKD) include baseline renal function, older age, and duration after kidney donation [51].

### 6.2. Decline in Kidney Function

The clinical course and risk factors for developing ERF after kidney donation have not been properly investigated [52]. Donor nephrectomy represents a sudden loss of approximately 50% of the nephron mass with an immediate and corresponding decrease in GFR; however, the remaining contralateral healthy renal parenchyma has the ability to recover a significant percentage of lost function within a relatively short period—as early as one month [53]. Velosa et al. [54] showed that as early as one week after nephrectomy, renal function has recovered to levels slightly lower than those achieved at six months after nephrectomy. Others have shown that the GFR at one year after donation was essentially similar to the value achieved at one week after donation [55, 56], suggesting little recovery of function after the initial period.

It has been observed that donors with a decreased renal mass may have a higher risk of developing proteinuria, hypertension, and chronic renal disease during long-term followup [51]. Saxena et al. [57] also examined the magnitude of adaptive hyperfiltration in the remaining kidney of 16 older (>57 yr) and 16 younger (<55 yr) individuals who had undergone a contralateral nephrectomy and concluded that the magnitude of adaptive hyperfiltration is similar in the elderly to that in young subjects with single kidneys, albeit at a lower absolute GFR level.

Velosa et al. [54] evaluated 140 donors (105 were <35 years and 35 were >55 years old) in whom the predonation GFR in the younger group of 113 mL/min was compared to 88 mL/min in the older group and showed that postnephrectomy percentage change of GFR was 68 ± 8 and 65 ± 8 in younger and older groups, respectively. This is similar to the finding in a larger prospective study (1994–2006) of 539 consecutive recipients of kidneys from 422 living donors <60 years, compared to 117 living donors >60 years, in which elderly donors had lower GFR before donation (80 versus 96 mL/min resp., *P* < 0.001) [58]. During a median follow-up of 5.5 years, the maximum decline in eGFR was 38% ± 9% and the percentage maximum decline was not different in both groups. On long-term followup, significantly more elderly donors had an eGFR <60 mL/min (131 (80%) versus 94 (31%), *P* < 0.001) [59, 60].

A study by Poggio et al. [61] of 1015 donors showed that the decline in GFR was approximately 4 mL/min per 1.73 m^2^ per decade of life for donors who were younger than 45 years, compared to 8 mL/min per 1.73 m^2^ in those older than 45 years. Several investigators hypothesized that kidneys from older donors would have a decreased “renal reserve capacity” that would manifest as impaired kidney function after donation [14].

Barri et al. [62] examined the effect of donor nephrectomy on GFR at 3 months and the occurrence of stage 3 CKD using I-iothalamate GFR (iGFR), modification of diet in renal disease (MDRD) estimated GFR, Cockcroft-Gault estimated creatinine clearance, and endogenous 24-hr creatinine clearance and found that the prevalence of stage 3 CKD was greater in the elderly. The long-term impact of stage 3 CKD after uninephrectomy is poorly understood and may not have the same implications as stage 3 CKD brought on by other causes [63].

### 6.3. ERF

Living donors have long-term risks that may not be apparent in the short term. The Japanese study of eight donors who developed ERF and were compared with a control population of 24 donors matched for age, sex, and follow-up time since donation showed that, apart from one donor who developed ERF caused by a traffic accident, none developed progressive renal dysfunction immediately after donation. However, after 10 years, the development of persistent proteinuria, cardiovascular event, or major infection heralded CKD [52]. The organ procurement and transplantation network (OPTN) examined its database, cross-checking it with renal waiting list history files to identify previous living donors subsequently listed for cadaveric kidney transplantation. They identified a total of 56 such people—43 having received transplants and two candidates had died while waiting [64]. A survey by OPTN in conjunction with the Centre for Medicare and Medicaid Services identified 126 cases of ERF among 56458 living kidney donors (0.22%), who donated during 1987–2003 [65]. The overall ERF risk was 0.134 per 1000 years at risk with an average duration of followup of 9.8 years. ERF rates for living donors overall and for Black, White, male, and female donors were compared favourably to the ERF incidence in the general population. The ERF rate in living donors was nearly five times higher for Blacks than for Whites and two times higher for males than females. In another report, which focused on African Americans, OPTN data revealed that, although African Americans comprised 14% of living kidney donors, they constituted 43% of former donors who were listed for transplantation [66]. However, these ethnic and gender-related differences were similar to those previously reported for ERF in the general population and support the current practice of living kidney donation [67]. Only three of 84 donors in a cohort of 464 living donors died with/from kidney failure [18].

### 6.4. Hypertension in Donors

El-Agroudy et al. conducted a retrospective analysis of 146 living-related donors >50 years old from 1976 to 2005 and reported that the rate of diabetes and hypertension was similar to that of an age matched general population [68]. Other reports including a larger Swedish study of 402 live donors found the age-adjusted prevalence of hypertension among donors to be similar to that in the general population [69]. However, a retrospective Norwegian study of 908 donors (1997–2007), showed a progressive increase in hypertension rate after kidney donation [70]. This increase in hypertension risk was also the conclusion of a meta-analysis concluded by the donor nephrectomy outcome research network, revealing that kidney donors may have a 5 mmHg increase in blood pressure within 5 to 10 years after donation over that anticipated with normal aging [71].

### 6.5. Quality of Life

The type of nephrectomy may exert important influence on the quality of life after donation. Laparoscopic nephrectomy is associated with less postoperative pain and better quality of life compared to open donor nephrectomy [72]. Kok and coworkers have reported that, one year after donation, patients who underwent laparoscopic nephrectomy had less physical fatigue and better level of physical function than those who had open donor nephrectomy [73].

Minnee and coworkers conducted a prospective study of postoperative complications and quality of life in 105 consecutive living donors who underwent a laparoscopic donor nephrectomy between 2002 and 2006, comparing donors over 55 years with younger donors [74]. They found no significant differences in intra- and postoperative complication rates even though elderly donors (*n* = 34) had both a significantly lower postoperative pain on day one (*P* = 0.019) and a lower total pain score in the analysis for the whole follow-up period (*P* = 0.002). The surgical outcome and quality of life were similar in both groups. Although their cut-off age for the elderly was relatively low at 55 years, their study gives support to the use of elderly donors in screening programs for transplantation. Chien et al. [75] and Shrestha et al. [76] also showed that the effect of donation on quality of life is not related to donor age or gender.

## 7. Outcome of Transplantation from Elderly Living Donors

### 7.1. Perioperative Complications

O'Brien et al. [9] performed a cross sectional study on 383 living donors stratified into groups according to age (<65 years, >65 years) and BMI in a single centre with followup of over 5 years and showed no significant differences in operative parameters such as operative time and estimated blood loss between groups. Although rates of early postoperative complications were not significantly different, subgroup analysis showed a higher incidence of respiratory complications at the extremes of obesity (body mass index ≥ 40 kg/m²). They concluded that nephrectomy in selected donors who may otherwise have been precluded on account of their age or weight resulted in perioperative or longer term outcomes comparable with their younger counterparts.

In a study comparing 115 recipients of kidneys from living donors >60 years with 158 from donors <60 years, the frequency of acute rejection (AR) episodes was found to be similar in both groups, but delayed graft function occurred more frequently in the former group [77]. The frequency of chronic renal allograft dysfunction in the first posttransplant year was significantly higher in transplants from older donors.

Ferrari et al. [78] assessed the impact of donor-recipient age difference on living donor kidney transplant outcomes by using a multivariate competing risks Cox model and showed that donor-recipient age difference was neither associated with increased risk of acute rejection within the first six months, nor with increased patient death, death-censored graft failure, or serum creatinine at five or 10 years. However, the European senior program (ESP) [63] has shown that allocating elderly donor organs to elderly recipients while resulting in shorter cold ischemia times and reduced DGF rates, it is associated with a 5–10% higher rejection rate. Graft and patient survival were not negatively affected by the ESP allocation policy when compared to standard allocation rules. In a series of 147 DCD recipients, Akoh and Rana demonstrated a significantly higher acute rejection rate in younger recipients of older DCD grafts in spite of better HLA mismatch profile [79]. With the increasing use of elderly donors, it would be interesting to see what effect this has on transplant outcomes of younger recipients of grafts from older living donors.

### 7.2. Graft Function

The outcome of transplantation of kidneys from living donors has been shown to be superior to those from deceased donors with regards to early graft function and patient survival, irrespective of the degree of mismatch [6, 80–83]. Kerr and coworkers conducted a univariate analysis of 1,126 consecutive transplants (1985–1995) and demonstrated that the graft survival of kidneys from older living donors is better than deceased donor kidneys from older donors and is comparable to deceased kidneys from younger donors [6]. With living donor kidneys, delayed graft function (DGF) is unusual, and long-term results are equivalent for both related and unrelated donor transplantation. A systematic review of transplant outcomes for recipients of living donor kidneys from 1980 to June 2008 showed that recipients of kidneys from older living donors (>60 years of age) have poorer 5-year patient and graft survival than recipients of kidneys from younger donors [84]. This meta-analysis included studies with varying levels of evidence, but recent studies have showed no difference in graft survival in transplantation from older versus younger donor kidneys [78, 85]. Chang et al. [86] showed that with the exception of recipients aged 18–39 years, who had the best outcomes with donors aged 18–39 years, living donor age between 18 and 64 years had minimal effect on allograft half-life (difference of 1-2 years with no graded association). This study however, had a relatively small number of living donors >60 years and did not account for death as a competing risk. A more recent retrospective cohort study [23] demonstrated significant differences in overall survival, and death censored survival and death with graft function in recipients stratified according to living donor age categories. They reported a 53% increase in the hazard for total graft failure in recipients of kidneys from donors >60 years compared to donors between 18 and 29.9 years.

In a large retrospective series of 73,073 first kidney-only transplant recipients in the United States between 1995 and 2003 [87], it was reported that the risk of graft loss with living donors of 55–64 years was similar to that with deceased donors <55 years. However, recipients from living donors over 65 years had a higher relative risk of graft loss (hazard ratio (HR) = 1.3, 95% CI: 1.1–1.7) and >70 years (HR = 1.7, 95% CI: 1.1–2.6). In a select group of recipients from 219 living donors over 70 years in the US, graft loss was significantly higher than matched 50- to 59-year-old live donor allografts (subhazard ratio 1.62, 95% confidence interval 1.16–2.28, *P* = 0.005) but similar to matched nonextended criteria 50- to 59-year-old deceased donor allografts (subhazard ratio 1.19, 95% confidence interval 0.87–1.63, *P* = 0.3) [88]. Gill and coworkers [8] evaluated 23,754 kidney transplantations performed in recipients 60 years and older of which 7,006 were living donors (1,133 were >55 years, and 5,873 were ≤55 years) showing that outcomes were best in younger living donor transplantations, followed by standard criteria deceased donor and older living donor transplantations.

Kidneys from older donors provide a statistically poorer outcome in transplant recipients [49, 89]. A multivariate analysis of 1,063 recipients revealed that the age of a living donor is an important determinant of long-term graft survival, particularly in younger recipients [49]. Rizzari et al. [90] analysed 1,632 recipients who underwent LD kidney transplantation between 1990 and 2009 in the US. Donor age more than 65 years, five to six HLA mismatches, DGF, and acute rejection were independent predictors of decreased patient and graft survival. Even after controlling for recipient age, donor age of more than 65 years remained a risk factor for a worse outcome. This is in agreement with a large UK retrospective study of 3,142 first adult kidney transplants from living donors [91].

However, a retrospective cohort study [60] showed no significant difference in total graft loss when transplants from older living donors (>60 years) were compared with younger donors—HR: 1.29 (0.80–2.08). Øien and coworkers performed a Cox regression analysis to estimate the association between different risk factors including donor age, HLA-DR mismatch, female gender, graft survival, and acute rejection episodes in a prospective cohort study of 739 first time living donor transplantation [92]. Their multivariate analysis further showed donor age >65 years was a risk factor for graft loss in all time periods after transplantation. However, graft survival was not affected by donor age above 50 years if recipients did not experience an early acute rejection episode. In their series, the incidence of acute rejection increased in recipients of grafts from donors >65 years (*P* = 0.009)—similar to the finding by Akoh and Rana in a series of recipients of DCD kidneys from elderly donors [79].

A study from Korea evaluated the outcomes from 269 living donors, in which 64 were expanded criteria living donors and 205 were standard criteria donors [85]. Their definition of an expanded criteria living donor included at least one of five criteria (age greater than 60 years, body mass index >30 kg/m^2^, history of hypertension, estimated GFR <80 mL/min, and proteinuria or microscopic hematuria) [93]. The recipients of organs from the expanded criteria living donor group showed a lower estimated GFR at one year after transplantation than the standard criteria group (66.9 ± 16.0 versus 58.3 ± 11.2; *P* < 0.001) although graft survival was not different (*P* = 0.518).

## 8. Conclusions

Most studies showed that kidneys from older donors had relatively lower graft function, increased rejection episodes and poor long-term graft survival compared to kidneys from younger donors. The prevalence of hypertension, established renal failure, and quality of life in donors is comparable to that of the general population. A multicentred, long-term, and prospective database, which is specifically aimed to address the outcomes of kidneys from elderly living donors is recommended. This review demonstrates that there is no clear definition or agreement on who should be regarded as an elderly living donor. Many of the studies cited have used different age cutoffs—50, 55, 60, and 65 years. Age stratification may be required in any future study to properly elucidate the effects of aging on kidney function and living donation.

This review has uncovered two areas of urgent need of study in relation to elderly living kidney donors. Firstly, there is a dearth of robustly conducted studies on quality of life of elderly donors. Given the rising trend of living kidney donation, particularly in the elderly, and the increasing complexity of dependency needs of an aging population, such a study will provide much needed information. Secondly, there is a clear need to analyse the outcome of parents to offspring living donor transplantations, particularly from donors over 65 years. Such transplantation involves donation of a relatively lower nephron dose, and it is therefore important to determine whether they perform better long-term than deceased organs from younger donors.
